# Comparative effectiveness of diabetes self-management education and support intervention strategies among adults with type 2 diabetes in Texas

**DOI:** 10.3389/fpubh.2025.1543298

**Published:** 2025-03-18

**Authors:** Marcia G. Ory, Gang Han, Chinelo Nsobundu, Keri Carpenter, Samuel D. Towne, Matthew Lee Smith

**Affiliations:** ^1^Center for Community Health and Aging, Texas A&M University, College Station, TX, United States; ^2^Department of Environmental and Occupational Health, School of Public Health, Texas A&M University, College Station, TX, United States; ^3^Department of Epidemiology and Biostatistics, School of Public Health, Texas A&M University, College Station, TX, United States; ^4^School of Global Health Management and Informatics, University of Central Florida, Orlando, FL, United States; ^5^Disability, Aging, and Technology Cluster, University of Central Florida, Orlando, FL, United States; ^6^Southwest Rural Health Research Center, Texas A&M University, College Station, TX, United States; ^7^Department of Health Behavior, School of Public Health, Texas A&M University, College Station, TX, United States

**Keywords:** diabetes, self-management, A1c, randomized clinical trial, comparative effectiveness

## Abstract

**Background:**

With approximately 1-in-10 Texas estimated to be living with Type 2 Diabetes Mellitus (T2DM), and the steadily rising healthcare costs associated with non-managed T2DM, efforts are needed to help patients manage their diabetes and avoid costly health consequences. While many diabetes self-management interventions and solutions exist to improve health among people living with T2DM, less is known about the relative effectiveness of these interventions based on their delivery format and when used in combination. The purpose of this study was to identify the effectiveness of three intervention modalities to reduce hemoglobin A1c (A1c) among Texans with T2DM living in rural and urban settings.

**Methods:**

A three-arm randomized controlled trial (RCT) was conducted from November 2020 through March 2022. The three modalities included: (1) asynchronous virtual education and support program with one-on-one follow-up counseling [i.e., virtual Making Moves with Diabetes (vMMWD)]; (2) technology-based education and support (i.e., TBES); and (3) combined modality where participants sequentially received vMMWD and TBES (i.e., combined). Data were collected at baseline and again at 3- and 6-month follow-up. Using an intent-to-treat analysis, constrained longitudinal data analysis models were fitted to identify and compare changes in A1c over time.

**Results:**

Findings demonstrate the positive effects of all three intervention modalities (i.e., vMMWD, TBES, and combined) to significantly reduce A1c among participants. Longitudinal analyses identified that initial reductions in A1c at 3-month follow-up were sustained at 6-month follow-up. Findings were consistent among rural- and urban-residing participants.

**Conclusion:**

This RCT highlights the universal benefits of self-paced virtual diabetes self-management interventions to reduce A1c among Texans with unmanaged T2DM. Such low-cost interventions may be widely applicable for different settings and populations.

## Introduction

Type 2 Diabetes Mellitus (T2DM) is a major clinical and public health issue worldwide and has been characterized as a “modern preventable pandemic” (1). In the last 20 years, the number of adults with T2DM has doubled as the United States (US) population continues to age and become more overweight and obese. An estimated 37 million adults have T2DM, with about 1-in-5 people being unaware they have the disease (2). Currently, diabetes is the eighth leading cause of death, and T2DM accounts for 90–95% of all diagnosed cases (3). Relative to those without T2DM, people with T2DM are at higher risk for heart disease, stroke, and other serious complications, such as kidney failure, blindness, and amputation of a toe, foot, or leg (3). Healthcare costs are twice as high for people with diabetes (~$327 billion annually) compared to those without diabetes (3).

In Texas, the growing and aging population continues to fuel increasing T2DM trends (4). The Centers for Disease Control and Prevention (CDC) approximates that 2.8 million adults in Texas have T2DM, which is equivalent to about 1-in-10 Texas residents (4). Texas spends $18.9 billion in direct medical costs and $6.7 billion in indirect medical costs related to T2DM (4). A recent study of commercially insured Texans identified that higher hemoglobin A1c (A1c) values and associated diabetes-related complications were diabetes-related cost drivers (5). These findings reinforce the importance of diabetes management strategies in community and clinical settings to regulate A1c levels and avoid preventable and costly complications.

Many diabetes self-management programs are available in the U.S. with a common goal of helping people with T2DM obtain the education, skills, and confidence to manage their diabetes in collaboration with healthcare providers (6–8). Diabetes Self-Management Education (DSME) programs are widely delivered and effectively enhance patients’ knowledge and skills necessary for sufficient self-care (9–11). These programs recognize the importance of patient-provider collaboration and the development of problem-solving skills for sustained self-care. Previous research has examined the effect of such programs on improving clinical outcomes like A1c (9, 11–33). Diabetes Self-Management Support (DSMS) includes activities that support the initiation and maintenance of healthful behaviors for ongoing diabetes self-management, including education, behavior modification, psychosocial and/or clinical support (34). DSMS activities are useful when used independently, but their impacts can be greater when used as complements to sustain the benefits of DSME (34).

There is growing recognition about the benefits of combining DSME and DSMS to create Diabetes Self-Management Education and Support (DSMES) programs, which are most advantageous for improving glycemic control, self-efficacy, and self-care behaviors, as well as reducing diabetes-related distress and foot complications (34, 35). DSMES programs are typically accredited by the Association of Diabetes Care & Education Specialists (ADCES) or recognized by the American Diabetes Association (ADA), which improves patient care and alignment with national standards for achieving population health goals (34). A DSMES program in Texas reported statistically significant reductions in A1c levels at 3 months that were sustained at 6-, 9- and 12-month follow-up assessments (8, 36). Although DSMES programs have demonstrated clinical and economic efficacy, these programs are typically underutilized resulting in a large research-to-practice gap (8, 36), thereby requiring innovative strategies to increase reach and utilization. Further, with the advent of COVID-19 and stay-at-home guidelines, DSMES programs typically delivered in small group, in-person formats were redesigned into virtual formats to maintain availability and accessibility (37).

Virtual DSMES programs provide an effective and time-efficient means of ensuring self-management supports and are delivered via multiple mediums and technologies including the internet, telephone, and text messaging (33–37). A recent meta-analysis also supports the efficacy of telemedicine for improving glycemic management and other health-related and quality-of-life outcomes (38). With a recent acceleration of diabetes-related digital health, there are now hundreds of smartphone applications for people with T2DM; however, few rigorous studies have examined their benefits in terms of clinical health outcomes (39).

While DSMES programs often have many positive documented benefits, programmatic attrition is often high (8, 36), which suggests the need for more engaging and interactive intervention approaches (8, 36). Further investigation is also needed to understand the relative effectiveness of different DSMES approaches to achieve meaningful diabetes-related benefits among people living with T2DM. In this context, the primary objective of this study was to identify the comparative effectiveness of a three-arm randomized controlled trial (RCT) among persons with T2DM in Texas living in rural and urban settings. More specifically, this study compared the efficacy of three intervention modalities: (1) asynchronous virtual education and support program with one-on-one follow-up counseling [i.e., virtual Making Moves with Diabetes (vMMWD)]; (2) technology-based education and support (i.e., TBES); and (3) combined modality where participants sequentially received vMMWD and TBES (i.e., combined). These three self-management modalities were tested in terms of pre-post changes on A1c values, and their efficacy was examined within rural and urban contexts.

We hypothesized that participants would generally benefit from any modality of the intervention received given that all adhered to best diabetes self-management practices; however, we anticipated that the combined modality would have superior effectiveness relative to either separate modality (i.e., vMMWD or TBES). Additionally, based on a deficit health access framework (40, 41), we hypothesized that persons from more disadvantaged backgrounds (e.g., younger adults without guaranteed Medicare health coverage, those with poorer baseline health, those living in rural areas, and those from underrepresented racial and ethnic groups) once exposed to health promotion programs would have the greatest intervention benefit reducing health inequalities.

## Methods

### Population and setting

**Figure 1:**
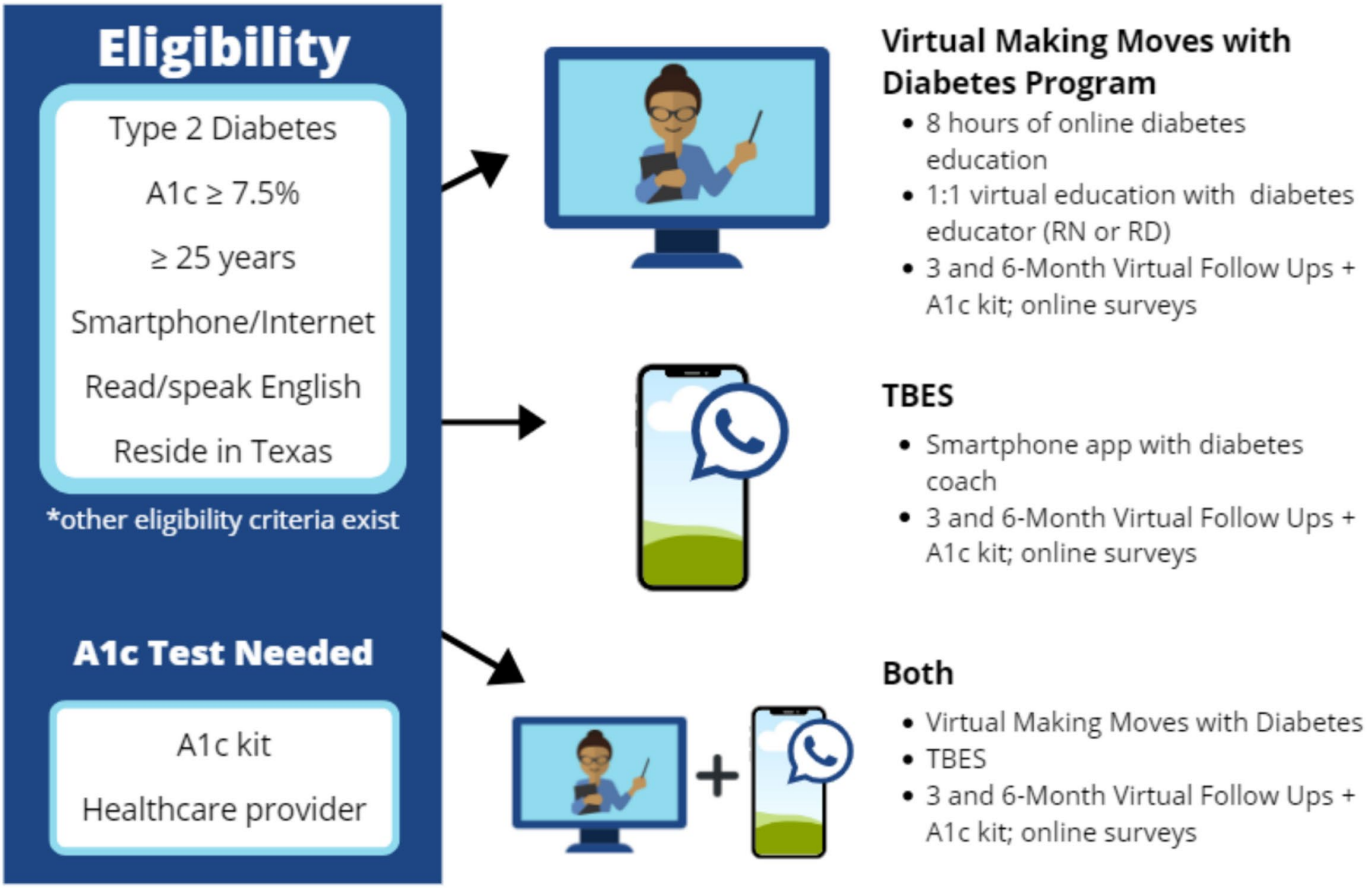
Study eligibility and intervention description.

The intended study population was adults aged 25 years and older, living in Texas with T2DM, with baseline A1c levels of 7.5 or higher (i.e., to indicate unmanaged T2DM and thereby potentially benefiting from intervention). The initial goal was to recruit participants from rural and urban areas of Texas, with approximately 50% residing in rural areas. To be eligible for the study, all participants needed to have access to a smartphone with an internet connection and be able to read and speak English (see Figure 1) for an intervention overview and eligibility criteria.

### Recruitment and retention strategies

**Figure 2:**
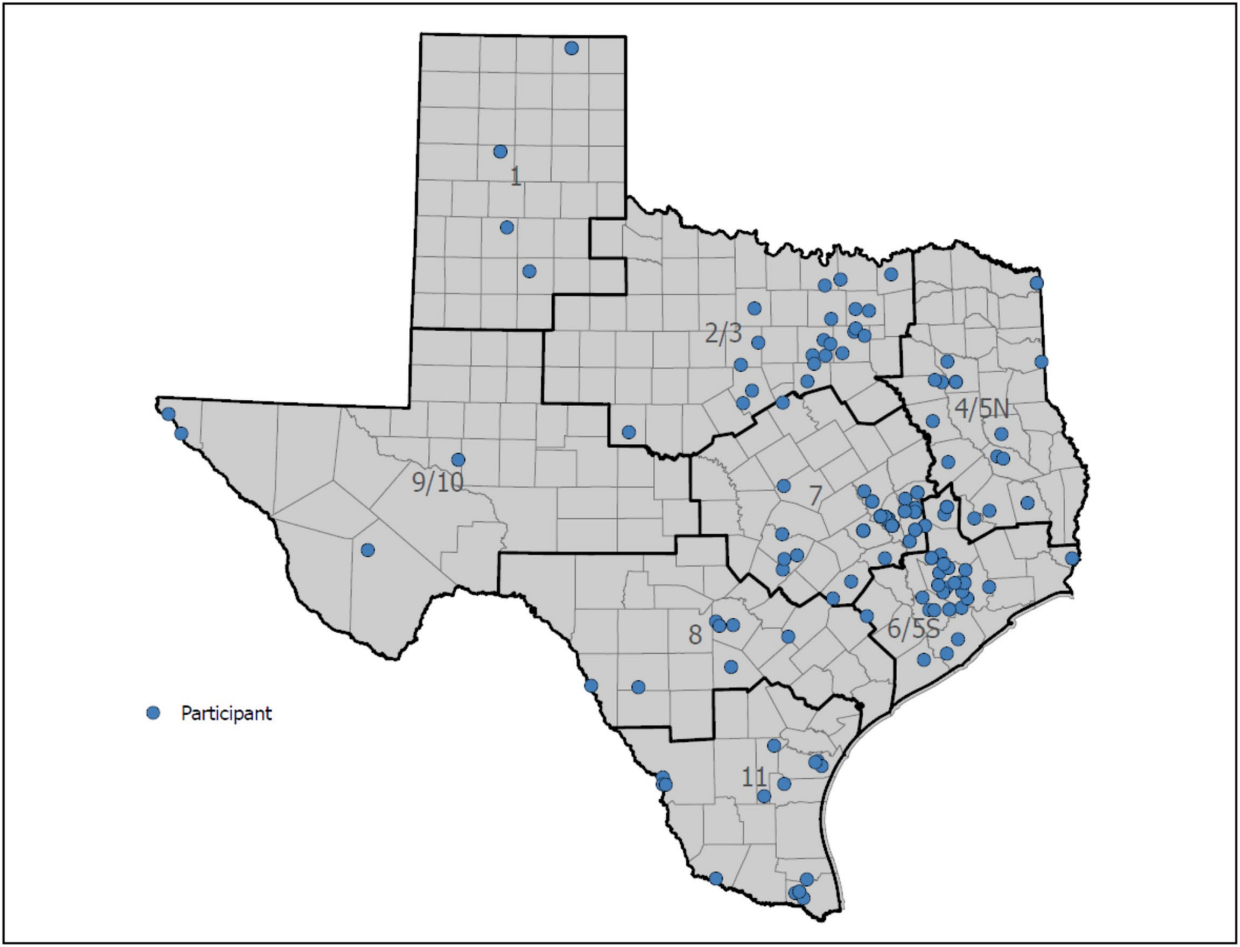
Participant reach across Texas by public health region.

Participants were enrolled in the study from November 2020 through March 2022. Study team members first consulted with key clinical, professional, and community members to identify potential recruitment opportunities and learn how to best recruit and retain participants residing in different communities. Using this information, the study team developed a recruitment plan to strategically reach participants through in-person and virtual means, with particular focus recruitment of participants residing within rural areas. By design, participant recruitment did not purposively target all 11 Public Health Regions in Texas. Rather, as described in detail below, recruitment strategies were primarily based on the intent to offer programming in-person prior to the COVID-19 pandemic. As a result, recruitment efforts yielded participants mostly from Central Texas, with expanded recruitment yielding representation across the state (see Figure 2).

The study team recruited participants utilizing radio advertisements, radio segments, flyers, bulletin boards, social media campaigns, directed letter campaigns from in-network healthcare providers, newspapers, email listserv, referrals (i.e., self and by family and friends), in-person recruitment, and the study’s website. Recruitment obstacles were encountered during the COVID-19 pandemic when many in-person interactions were limited in key clinical, professional, and community settings. In clinical settings, the study team met in-person and virtually with clinicians and healthcare personnel at hospitals and nonprofit healthcare clinics to describe the study. Key partners were given recruitment letters and flyers, which outlined the study’s purpose, eligibility criteria, incentives, and protocol. The study team received access to participant names and phone numbers for those who were interested in, and potentially eligible for, the study. Participants could also self-refer and contact the study team to determine eligibility. While in-person recruitment was limited at different times during the pandemic, study team members were able to recruit in the welcome areas of local clinics (i.e., talk to interested participants and offer an HbA1c eligibility test after completing an eligibility screener). To boost initial low recruitment, in-person meetings with healthcare professional staff, administrative staff, and clinicians helped to increase professional and self-referrals from healthcare settings.

Professional and community sites (e.g., small businesses, community events, churches, outdoor and virtual health fairs, local food pantries, recreation centers, senior centers, community health resource centers, and educational events) were effective recruitment strategies because of limited in-person access to clinical sites during the COVID-19 pandemic. Community liaisons, executive directors, and other community leaders were important partners and stakeholders who helped raise awareness about the study. At in-person events, the study team was available to answer questions during the eligibility process and offer an HbA1c eligibility test for any interested participants. Additionally, interested participants contacted the study team to determine eligibility.

Over the course of the study, best practices were also used to enhance participant retention rates (42–44). Knowing the participant recruitment challenges encountered by DSMES community initiatives in Texas (8, 36), the study team bolstered retention by using participant incentives, distribution of study information, and frequent communication with the administrative study team via text messaging, emails, and phone calls for intervention guidance, recognition of milestones in the intervention, birthday messaging, study visit reminders, and other periodic touchpoints. Participants received electronic gift cards, which were staggered over the intervention period from initial screening through each follow-up assessment. Overall, depending on their participation in the study, each participant could be given upwards of $375 for completing data throughout the study period.

### Research design and randomization

**Figure 3:**
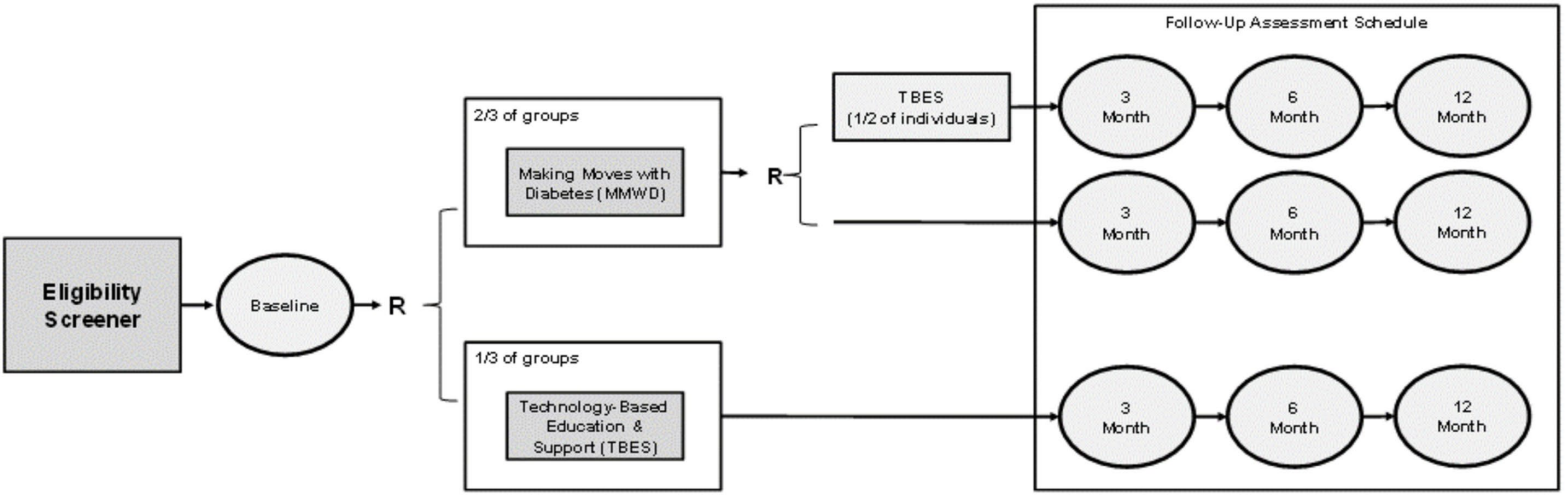
Two-stage randomization scheme.

As further illustrated in Figure 3, to examine study aims, a RCT was designed and conducted to assess the comparative effectiveness of three intervention modalities: (1) vMMWD only; (2) TBES only; and (3) combined modality where participants sequentially received vMMWD and TBES. Each of the three intervention modalities were employed for 3 months, with uniform data collection at baseline and follow-up periods at 3 months and 6 months. The study team designed a two-stage randomization plan to assign participants into an intervention arm as illustrated in Figure 3. Power was calculated for the primary outcome (i.e., A1c) prior to initiating the study. The anticipated A1c change was identified in data from a previous study using a similar intervention in South Texas (8, 36). Assuming a moderate effect size (i.e., Cohen’s D of 0.5), we calculated that we would need 34 participants from baseline and post intervention to detect significant changes in A1c with 80% power at significance level 0.05 using two-sided paired t-test.

The first stage of randomization was an individual-based block stratified randomization into one of two arms: vMMWD only or TBES only. This randomization included a ratio of 2:1 where 2 participants were assigned to receive vMMWD relative to 1 participant assigned to receive TBES. The randomization unit was each individual, and eligible participants in the same family or household were placed in the same intervention arm if they were close family members, including spouses, partners, siblings, and parents (i.e., to avoid contamination effects within a household). The randomization into the vMMWD and TBES arms was executed for residents residing in rural and urban areas separately as the two strata. The block of size 3 was used for the block randomization across different study sites and across different groups in each study site. Then, among the participants who completed vMMWD in stage one, the second stage of randomization was an individual-based block randomization for one of two arms: vMMWD only (i.e., no additional intervention) or combined modality (i.e., receive TBES after they already received vMMWD). This randomization included a ratio of 1:1.

**Figure 4:**
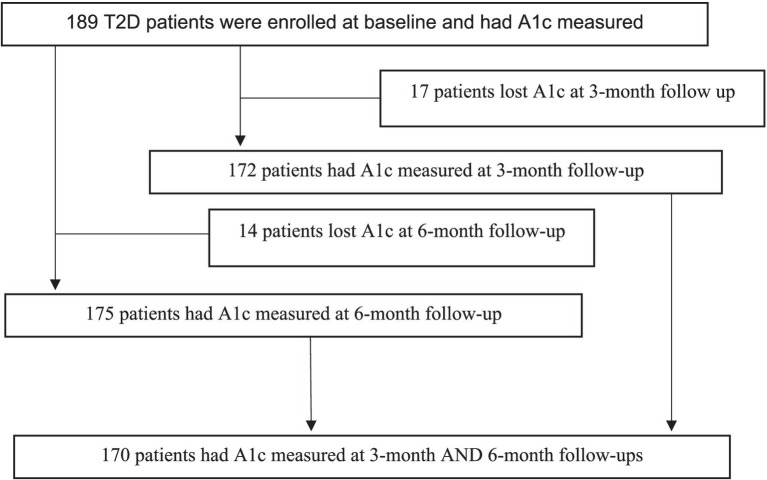
Patient flow diagram.

After eligibility was determined, 189 participants with T2DM were enrolled in the study across 46 Texas counties between November 2020 and March 2022. Figure 4 provides a visualization of our enrollment and retention flow for this study.

Of the 189 participants who completed baseline assessments, approximately 90% of enrollees had data at both follow-up time points. All participants provided written consent to participate in this RCT. Participation was voluntary and participants could withdraw from the study at any time without penalty. The current study was reviewed and approved by the Texas A&M Institutional Review Board (IRB #2019-0804D). Deidentified data on major study outcomes will be placed in the Texas A&M data repository upon completion of data analyses and report writing. This clinical trial is registered at ClinicalTrials.gov under ID number NCT06370494.

### Intervention strategies

Three community-based DSMES intervention strategies were tested under a Living Healthier with Diabetes program. Each one was aligned with American Diabetes Association (ADA) national standards for diabetes self-management and best behavioral change practices but had different delivery modalities (34). As illustrated in Figure 1, the three interventions were: (1) Virtual asynchronous training with periodic one-on-one counseling with a diabetes specialist (vMMWD Group); (2) Smartphone application for more continuous but less structured support (TBES Group); and (3) a combined approach for maximal support and reinforcement (Combination Group).

The vMMWD Group participated in three education engagements over 3 months. vMMWD was comprised of 6-to-8 h of virtual asynchronous training, which were complemented by one-on-one interactions between participants and either a registered nurse or registered dietician. During these personalized one-on-one education and counseling sessions, participants received individualized attention to build upon what was learned during the asynchronous training and create tailored strategies to optimize their diabetes management and self-care.

The TBES Group had access to a mobile application, which they could use at their convenience to learn and practice diabetes self-care skills and strategies. In addition to the built-in features of the mobile application, the TBES included a chat feature with a diabetes coach, which allowed each participant to get tailored support and have a personalized experience.

The Combination Group had sequential access to both intervention types. Participants in this randomized condition first completed vMMWD, then were provided access to TBES. Participants in all three intervention groups received an A1c monitoring kit, a glucose meter, and supplies for testing their blood sugar within their own homes.

### Measures

#### Dependent variable

Hemoglobin A1c (A1c) measures the average plasma glucose in the previous 3 to 3 months (45). Each participant had their A1c values measured at baseline, 3-month follow-up, and 6-month follow-up. Participants in each group received A1c monitoring kits and glucose meters and supplies for testing in their homes [i.e., the A1cNow + kit from PTS Diagnostics (46)]. A1c values were treated continuously in analyses, with possible values ranging from 4 to 14 due to instrument precision at the higher values. In addition to the continuous A1c measurements, an indicator variable was created to indicate a clinically meaningful change (e.g., whether A1c reduced by more than 0.5 at the 6-month).

#### Rurality

The National Center for Health Statistics (NCHS) Urban–Rural Classification Scheme for Counties was utilized to assess potential variation across metropolitan and non-metropolitan areas (47). This measure has been used to discuss levels of rurality throughout the published literature (47–49). While there are varying definitions of what constitutes a rural area, the Federal Office of Rural Health Policy (FORHP) considers “non-Metro counties as rural,” (48) while the Office of Management and Budget (OMB) indicates that Micropolitan counties are defined as “non-Metropolitan or rural” (48). For the purposes of this study, we note that these measures often constitute a continuum from the most metropolitan areas to more rural areas but refer to urban areas as those defined as metropolitan (NCHS Urban–Rural Classification: 3–6) and rural areas as those defined as non-metropolitan (NCHS Urban–Rural Classification: 5 and 6 for Micropolitan and Non-Core, respectively).

#### Self-rated health status

At baseline, participants were asked to self-report their health status by answering the following question (50): “Would you say that in general, your health is: excellent, very good, good, fair, and poor.” This variable was trichotomized based on the response distribution: “poor/fair,” “good/very good,” and “excellent.” Higher scores indicated better self-reported health status.

#### Socio-demographic characteristics

Participant characteristics included in analyses were age (measured continuously by year of birth and also displayed by 10-year groupings), sex (female, male), education (high school or less, more than high school), ethnicity (Hispanic/Latino, Non-Hispanic/Latino), and race (White, Black or African American, American Indian or Alaska Native, Asian, Native Hawaiian or Other Pacific Islander). The race variable was dichotomized given the response distribution (White, Non-White). Financial stability was defined as having ends meet at the end of each month, which was dichotomized as “yes” or “no.” Participants were also asked to report if they currently had insurance coverage, which was dichotomized as “yes” (any type of insurance coverage) and “no” (no insurance).

### Statistical analysis

An intent-to-treat (ITT) analysis was conducted so that all participants who enrolled were included in the analysis and analyzed based on the arms to which they were randomized (51). This ITT analysis was in compliance with the CONSORT statement and employed because it can preserve the baseline balance of the arms and prevent selection bias and confounding (52, 53). Descriptive statistics were calculated for all participants, by intervention arms (vMMWD only, TBES only, combined), and by rurality (rural, urban). Medians with interquartile ranges (IQR) are reported for continuous variables, and frequencies with percentages are reported for discrete variables. Wilcoxon rank-sum tests, Kruskal-Wallis tests, and Pearson’s Chi-square tests were used for testing continuous and categorical variables, respectively.

Changes in A1c were calculated as differences between A1c at baseline and A1c at 3-month follow-up, and between A1c at baseline and A1c at 6-month follow-up. The average changes and 95% confidence intervals (CIs) of the mean changes based on t-distributions were calculated for all participants, for participants in each study arm, and for participants by rural and urban areas.

Longitudinal A1c measurements at 3-month and 6-month follow-ups were modeled using intervention arm, demographic variables, rural status, and self-reported health status. The constrained longitudinal data analysis (cLDA) model proposed by Liang and Zeger (54) was used where both baseline and follow-up measures were treated as dependent variables, and the baseline means from three treatment arms were constrained to be the same across the treatment groups due to the randomization. According to the literature (55, 56), cLDA models can lead to more efficient and robust estimation than other models, such as analysis of covariance and unconstrained longitudinal data analysis. In addition to the continuous A1c measurements, an indicator variable was created to label whether A1c reduced by more than 0.5 at the 6-month follow-up, which indicates a clinically meaningful A1c reduction (57). Odds ratios and 95% CI were estimated in the longitudinal analysis using a generalized linear model with repeated measurements. All analyses were conducted using SAS software, version 9.4 (SAS Institute, Cary, NC). The longitudinal analysis was implemented using SAS software, procedure GENMOD. A *p*-value of 0.05 or less was considered statistically significant.

## Results

Tables 1, 2 shows descriptive statistics for all participants, participants by each intervention arm, and participants by rural and urban residence. The median age of participants was 52 years, with an IQR of 44–59 years. In terms of grouping by age category, younger adults (ages 25–49) comprised 39.68% of study; middle-aged adults (ages 50–64) comprised 46.56% of study; and older adults (ages 65 plus) comprised 13.76% of population. Twenty-three percent of participants were men and 35% resided in rural areas. Most participants were White (86%) and non-Hispanic/Latino (61%), and 55% had “poor/fair” self-reported health. The median A1c value at baseline was 8.9 and the IQR was of 8.3–10.3. The median A1c value was 7.9 at 3-month follow-up and 8.0 at 6-month follow-up. No significant differences were observed across the three intervention groups. In terms of rural vs. urban residence, a significantly larger proportion of Hispanic/Latino participants resided in urban areas (44%) than in rural areas (28%), with a *p*-value of 0.03. In terms of other sociodemographic variables, 21% had a high school education or less; 15% reported problems making ends meet every month; and 14% had no insurance coverage. Financial stability, education, and insurance were not significantly associated with intervention arm or rurality, respectively.

**Table 1:** Descriptive statistics by intervention arm.

Variable	Level	App only	Class only	Combined	*p*-value
Age		53.5, [46.5, 59.5] *N* = 68	50, [43, 58] *N* = 60	53, [43, 59] *N* = 61	0.27
Sex	Female	54 (79)	47 (78)	45 (74%)	0.73
	Male	14 (21%)	13 (22%)	16 (26%)	
Ethnicity	Hispanic/Latino	23 (34%)	25 (42%)	25 (41%)	0.59
	Not Hispanic/Latino	45 (66%)	35 (58%)	36 (59%)	
Binary race	Non-White	7 (10%)	11 (18%)	8 (13%)	0.41
	White	61 (90%)	49 (82%)	53 (87%)	
General health	1. Poor/fair	36 (53%)	33 (55%)	35 (57%)	0.17
	2. Good/very good	27 (40%)	22 (37%)	15 (25%)	
	3. Excellent	5 (7%)	5 (8%)	11 (18%)	
A1c baseline		9.2, [8.3, 10.5] *N* = 68	8.8, [8.3, 10] *N* = 60	8.8, [8.2, 10.6] *N* = 61	0.39
A1c at 3 months		7.7, [7, 8.9] *N* = 58	7.8, [6.8, 8.9] *N* = 57	8.3, [7.3, 9.4] *N* = 57	0.27
A1c at 6 months		8, [7.3, 9.1] *N* = 60	8, [6.8, 8.9] *N* = 58	7.8, [7, 9.1] *N* = 57	0.67
Rural status	Rural	23 (34%)	22 (37%)	22 (36%)	0.94
	Urban	45 (66%)	38 (63%)	39 (64%)	
Financial stability	0. No	10 (15%)	10 (17%)	9 (15%)	0.94
	1. Yes	58 (85%)	50 (83%)	52 (85%)	
Education	High school or less	14 (21%)	9 (15%)	16 (26%)	0.31
	More than high school	53 (79%)	51 (85%)	45 (74%)	
Insurance covered	1. Yes	50 (86%)	50 (86%)	47 (85%)	0.99
	2. No	8 (14%)	8 (14%)	8 (15%)	

Median [interquartile range] reported for continuous variables; frequency (percentage) reported for categorical variables.

**Table 2:** Descriptive statistics for all and by rural status.

Variable	Level	All	Rural	Urban	*p*-value
Age		52, [44, 59] *N* = 189	52, [43, 58] *N* = 67	51.5, [44, 59] *N* = 122	0.81
Sex	Female	146 (77%)	53 (79%)	93 (76%)	0.65
	Male	43 (23%)	14 (21%)	29 (24%)	
Ethnicity	Hispanic/Latino	73 (39%)	19 (28%)	54 (44%)	0.03
	Not Hispanic/Latino	116 (61%)	48 (72%)	68 (56%)	
Binary race	Non-White	26 (14%)	6 (9%)	20 (16%)	0.16
	White	163 (86%)	61 (91%)	102 (84%)	
General health	1. Poor/Fair	104 (55%)	36 (54%)	68 (56%)	0.91
	2. Good/very good	64 (34%)	24 (36%)	40 (33%)	
	3. Excellent	21 (11%)	7 (10%)	14 (11%)	
A1c baseline		8.9, [8.3, 10.3] *N* = 189	8.6, [8.3, 9.8] *N* = 67	9.1, [8.3, 10.4] *N* = 122	0.34
A1c at 3 months		7.9, [7.1, 9.3] *N* = 172	8.3, [7, 9.4] *N* = 62	7.7, [7.1, 9.1] *N* = 110	0.46
A1c at 6 months		8, [6.9, 9] *N* = 175	8.2, [7.2, 8.9] *N* = 64	7.9, [6.8, 9.1] *N* = 111	0.43
Study arm	App Only	68 (36%)	23 (34%)	45 (37%)	0.94
	Class Only	60 (32%)	22 (33%)	38 (31%)	
	Combined	61 (32%)	22 (33%)	39 (32%)	
Financial stability	0. No	29 (15%)	10 (15%)	19 (16%)	0.91
	1. Yes	160 (85%)	57 (85%)	103 (84%)	
Education	High school or less	39 (21%)	17 (25%)	22 (18%)	0.24
	More than high school	149 (79%)	50 (75%)	99 (82%)	
Insurance covered	1. Yes	147 (86%)	53 (84%)	94 (87%)	0.60
	2. No	24 (14%)	10 (16%)	14 (13%)	

Median [interquartile range] reported for continuous variables; frequency (percentage) reported for categorical variables.

Table 3 reports the point estimates and 95% CIs of the change of A1c between baseline and follow-up assessments. On average, A1c values significantly decreased from baseline to 3-month and 6-month follow-ups for all participants, participants in each of the three intervention groups, and participants living in rural and urban areas, respectively (*p* < 0.001). This indicates all three intervention modalities had continuous effects of reducing A1c in rural and urban areas. Although not statistically significant, changes from baseline to 6-month follow-up were on average equal (for TBES only) or higher (for rural, urban, vMMWD only, combination) than the changes from baseline to 3-month follow-up, according to Table 3. This indicates that A1c could continue to drop after 3-month follow-up.

**Table 3:** Mean difference of A1c change at follow-up assessments (or average A1c reduction).

A1c	Baseline to 3-month follow-up	Baseline to 6-month follow-up
Total	1.09, (0.83, 1.35)**; *N* = 172; *D* = 0.631	1.21, (0.98, 1.44)**; *N* = 175; *D* = 0.797
Rural	1.04, (0.55, 1.53)**; *N* = 62; *D* = 0.526	1.11, (0.73, 1.49)**; *N* = 64; *D* = 0.714
Urban	1.12, (0.82, 1.42)**; *N* = 110; *D* = 0.709	1.26, (0.98, 1.54)**; *N* = 111; *D* = 0.845
TBES Only	1.31, (0.80, 1.82)**; *N* = 58; *D* = 0.663	1.31, (0.91, 1.71)**; *N* = 60; *D* = 0.839
vMMWD Only	1.00, (0.54, 1.46)**; *N* = 57; *D* = 0.571	1.11, (0.73, 1.49)**; *N* = 58; *D* = 0.743
Combination (vMMWD + TBES)	0.96, (0.59, 1.33)**; *N* = 57; *D* = 0.677	1.2, (0.81, 1.59)**; *N* = 57; *D* = 0.797

95% confidence interval of the mean difference, and available sample size *N*, from baseline to 3 and 6 months for total, by rural status, and by arms. Statistical significance from testing if the A1c reduction was significantly different from 0 is indicated using “*” if the *p*-value is between 0.001 and 0.05, and “**” if the *p*-value is less than 0.001. D is the effect size, Cohen’s d for one sample t-test.

Tables 4, 5 reports the constrained longitudinal analysis results for changes in continuous A1c values (Table 4) and whether A1c reduced by more than 0.5 (Table 5). The continuous A1c value was significantly and negatively associated with follow-up time. On average, A1c reduced by about 0.5 for each follow-up period of 3 months. When assuming a linear change, on average A1c reduced by 0.169 for each month during the follow-up period (*p* < 0.0001). On average, older age was associated with lower A1c indicating better diabetes care for older patients than younger patients (estimate = −0.028, *p* = 0.007). Compared with those reporting “poor/fair” health at baseline, those reporting “good/very good” health had lower A1c by 0.318 on average (*p* = 0.032). Participants with financial stability had significantly lower A1c than those not (estimate = −0.857, *p* = 0.003). Higher education was associated with lower A1c (estimate = −0.762, *p* = 0.004). In the analysis of binary A1c dropped more vs. less than 0.5 (Table 5), the follow-up time remained significant (odds ratio estimate: 1.124, 95% CI: 1.102–1.147, *p* < 0.0001), indicating clinically significant improvement of A1c over time. However, no significant differences were observed between intervention arms in Table 4 or Table 5. The regression analysis indicates that A1c significantly reduced, during the 6 months follow-up period, in all three intervention groups.

**Table 4:** Results from longitudinal generalized linear regression of A1c.

Parameter	Levels	Estimate	95% CI		*p*-value
Follow-up time		−0.169	−0.232	−0.105	<0.0001
Age		−0.028	−0.048	−0.008	0.007
Sex	Female vs. Male	−0.122	−0.529	0.286	0.559
Ethnicity	Not Hispanic/Latino vs. Hispanic/Latino	−0.075	−0.467	0.317	0.708
Race	White vs. Non-White	−0.255	−0.838	0.329	0.392
Rural status	Urban vs. Rural	−0.027	−0.372	0.318	0.877
General health	Good/very good vs. Poor/Fair	−0.318	−0.609	−0.027	0.032
	Excellent vs. Poor/Fair	−0.297	−0.795	0.201	0.242
Financial stability	Yes vs. No	−0.857	−1.427	−0.287	0.003
Education	More than high school vs. High school or less	−0.762	−1.282	−0.242	0.004
Insurance covered	Yes vs. No	0.245	−0.315	0.806	0.391
Arm * time	Combined vs. App	−0.006	−0.091	0.078	0.882
	Class vs. App	−0.068	−0.164	0.027	0.162

The outcome variable is A1c measured at 3- and 6-month follow-ups.

**Table 5:** Results from longitudinal logistic regression of A1c change.

Parameter	Levels	Estimated odds ratio	95% CI of the odds ratio		*p*-value
Follow-up time		1.124	1.102	1.147	<0.0001
Age		1.001	0.997	1.005	0.696
Sex	Female vs. Male	0.982	0.893	1.079	0.701
Ethnicity	Not Hispanic/Latino vs. Hispanic/Latino	0.985	0.894	1.086	0.767
Race	White vs. Non-White	1.032	0.906	1.175	0.639
Rural status	Urban vs. Rural	1.047	0.957	1.146	0.315
General health	Good/very good vs. Poor/Fair	1.001	0.919	1.091	0.977
	Excellent vs. Poor/Fair	1.027	0.910	1.158	0.669
Financial stability	Yes vs. No	0.974	0.857	1.107	0.688
Education	More than high school vs. High school or less	1.026	0.925	1.139	0.623
Insurance covered	Yes vs. No	0.912	0.813	1.023	0.116
Arm * time	Combined vs. App	0.988	0.959	1.017	0.420
	Class vs. App	0.987	0.958	1.017	0.401

The outcome variable is A1c dropped more vs. less than 0.5 at 3- and 6-month follow-ups. The outcome variable is A1c measured at 3- and 6-month follow-ups.

## Discussion

### Principal results

This 3-arm RCT demonstrated the positive effects of a virtual asynchronous diabetes education with personalized counseling by professionals (i.e., vMMWD) and a self-guided smartphone application (i.e., TBES) to reduce A1c values among Texans with T2DM. These findings support other studies showing the effectiveness of similar diabetes self-management interventions on reductions in A1c values (9, 11–33). However, generally, the observed mean differences in A1c values and associated effect sizes in this study were quite large and comparatively better than other diabetes self-management interventions (9, 11–33). Further, the longitudinal analyses in the current study support the sustained A1c level reduction over time, in contrast to other intervention studies that show a diminution of effect over time (8, 36).

Per study hypotheses, on average, participants receiving any intervention modality reduced their A1c; however, no superiority effect was observed for participants receiving combined intervention modalities (i.e., vMMWD + TBES). While it was expected that a multilevel intervention that combined virtual training with personalized counseling and an on-demand educational TBES approach evoke larger intervention impacts given the multi-pronged educational programming and more intensive support and reinforcement (58) no significant differences were observed in this study.

The improvements in A1c among participants in both groups may indicate that vMMWD and TBES were both initially effective in evoking behavior change principles and promoting self-management thereby reducing A1c levels. Because all participants received vMMWD prior to receiving TBES in the combination arm of the trial, it is plausible that these participants already received the initial benefits from vMMWD before receiving TBES. Therefore, TBES in this instance may have served as a booster intervention to maintain newly acquired vMMWD skills and sustain initial A1c reductions. However, unfortunately, this study was unable to track the extent to which participants logged into the TBES, the duration spent in the mobile application, and/or the features utilized when using the mobile application. The absence of a superiority effect between intervention arms may also suggest that various interventions provided to people with T2DM can be equally effective as long as they adhere to recommended diabetes self-management programming, and T2DM patients can choose DSMES intervention modalities they prefer without a significant loss of effectiveness.

This study’s findings did not support the hypothesized deficit health access framework. The study team assumed that the availability of high-quality diabetes self-management that adhered to ADCES guidelines (34) could have differential positive effects on those who characteristically experienced higher health inequities in access to healthcare (i.e., by age, ethnicity, race, financial stability, education, insurance coverage and poorer self-reported health). However, this was not supported by study results. While it was expected that younger adults with less access to universal healthcare coverage would display greater changes than older adults who have more interactions with the healthcare system (59, 60) findings indicate that older adults tended to have larger reductions in A1c values and better diabetes management relative to the younger participants in this study. Although, this finding is not surprising given that older adults have been shown to reduce their A1c values by participating in diabetes self-management interventions (61).

We also assumed that access to quality diabetes self-management interventions would benefit those in poorer health more than their counterparts with a better health status since poor health status can reflect a lack of health care and access to health promotion interventions (62–64). Contrary to this assumption, those with better baseline health tended to have more positive outcomes in our study. This may be because a base level of good health is needed to proactively engage in the different diabetes self-care activities (65).

Rurality is typically characterized by a lack of healthcare access and inequities in knowledge about evidence-based diabetes self-management compared to their urban counterparts (66, 67). However, on average, both rural and urban residents exhibited significantly reduced A1c values over time, and no significant differences were observed between urban and rural participants in terms of these improvements. This suggests that the vMMWD and TBES interventions tested in this study are suitable for dissemination in rural settings. It should be noted that although this study used the NCHS designation to distinguish between rural (non-metropolitan) and urban (metropolitan) areas, other operational definitions of rural areas [by counties (68), Census Tracts (69), and otherwise (70)] are available and should be considered in future studies. Notably, the inclusion of rurality into this RCT is an important strength of this study because these underserved rural residents are often underrepresented in RCT. Because rural populations are a recognized health disparity population, as defined by the National Institutes of Health (71) (findings from this study are relevant to a wide group of stakeholders throughout the U.S.).

We also hypothesized that those in underrepresented groups, characterized by minoritized race or ethnicity, financial instability, lower education, or no insurance coverage, would also have less healthcare access and would benefit differentially if such programming was available. While race, ethnicity, rurality, and insurance coverage were not associated with intervention outcomes in terms of absolute changes in A1c, those with financial stability and higher education levels had greater improvements. In contrast, the sociodemographic factors reflecting non-medical drivers of health were not associated clinically meaningful changes in A1c values. Expected differences by race/ethnicity, financial stability, education level, or insurance coverage did not emerge. These findings suggest that underrepresented populations can benefit equally from self-management programs, and that future programs need to make an effort to reach such populations (72, 73).

While previous studies reported high attrition rates in community-based diabetes self-management initiatives (8, 36), the current study had approximately 90% retention at the 3-month and 6-month follow-up, respectively, as indicated in Figure 4. This high retention rate was likely attributed to the study team’s efforts to adhere to basic implementation research principles such as adapting the programs to fit the context, considering the feasibility of the selected interventions, and fostering participant engagement through interactive learning (74, 75).

### Limitations

While this study has many strengths, some limitations must be acknowledged. This study had a relatively small sample, which recruited participants from one state. Therefore, caution must be taken when attempting to generalize findings to different settings and populations. Recruitment strategies were primarily rooted within Central Texas, utilizing existing partners and social media in response to COVID-19. Therefore, the study sample may not have yielded a representative sample of adult Texans living with diabetes. Recruitment and intervention materials were only available in English, which may have influenced participation, especially in a state like Texas that has a diverse and multilingual population. It is possible that the technology-based approaches in this trial introduced participation bias. While the disparity gap in technology use/internet connectivity is narrowing in the United States, disparities still exist regarding access to, and comfort with, technology across different socioeconomic and urban/rural residences (76). Thus, while digital technology interventions have the benefit of reaching dispersed populations (77), such interventions may inadvertently increase disparity gaps (78).

The current study was conducted during the COVID-19 pandemic, making recruitment more difficult and necessitating new study protocols. The pandemic required a switch from in-person to virtual training for Making Moves with Diabetes, which may have inadvertently diminished the observable differences between the two main intervention strategies. However, the pandemic also facilitated innovative ways of collecting A1c remotely (i.e., sending self-administered kits to participant’s homes) for participants that did not have a recent A1c values from their healthcare providers. The original study intent was to additionally collect data at 12-months of follow-up, but the delayed start because of the COVID-19 pandemic only allowed the study team to collect 6 months of data on the full sample. Future studies should plan for longer follow-up periods to assess the long-term impact of diabetes self-management strategies.

This study narrowly focused on A1c as the only outcome, despite collecting multiple outcomes related to diabetes self-management, skill development, routine screening, and other health indicators. This study’s deliberate focus on A1c stems from the primary objective of the RCT to reduce unmanaged diabetes, on which the study design was powered. Future studies will expand the investigative scope by examining the influence of these intervention approaches on other behavioral and psychosocial variables (e.g., diabetes knowledge, efficacy to manage diabetes, and distress), which have also been shown to impact self-care behaviors and clinical outcomes. While this 3-arm RCT showed significant effects within each intervention arm, there was no true control group in this comparative effectiveness trial, which limited the ability to determine the degree to which these intervention approaches were effective relative to the absence of an intervention.

## Conclusion

Findings from this RCT support that diabetes self-management interventions that adhere to ADA/ADCES guidelines can successfully reduce unmanaged A1c values among T2DM patients. Due to the universal benefits received from participants across rurality and participant characteristics in this study, similar benefits can be expected among T2DM patients who receive (or choose to receive) virtual training with personalized counseling and/or a technology-based education and support (via a smartphone application). The positive clinical outcomes observed may be widely applicable to different settings and populations including those from underrepresented populations and individuals living in rural settings.

## Data Availability

The datasets presented in this article are not readily available because the data that support the findings of this study are available from Blue Cross Blue Shield of Texas but restrictions apply to the availability of these data, which were used under license for the current study, and so are not publicly available. Requests to access the datasets should be directed to Texas A&M point of contact for all data (Mr. Jim Colson, Texas A&M Vice President, Digital Health, jim.colson@tamu.edu).
